# Pirfenidone as a novel cardiac protective treatment

**DOI:** 10.1007/s10741-021-10175-w

**Published:** 2021-10-20

**Authors:** Alberto Aimo, Giosafat Spitaleri, Giorgia Panichella, Josep Lupón, Michele Emdin, Antoni Bayes-Genis

**Affiliations:** 1 Institute of Life Sciences, Scuola Superiore Sant’Anna, Pisa, Italy; 2 Fondazione Toscana Gabriele Monasterio, Pisa, Italy; 3 Heart Failure Clinic and Cardiology Service, University Hospital Germans Trias I Pujol, Badalona, Spain; 4 Department of Medicine, Universitat Autonoma de Barcelona, Barcelona, Spain; 5 CIBERCV, Instituto de Salud Carlos III, Madrid, Spain

**Keywords:** Pirfenidone, Fibrosis, Inflammation, Myocardial diseases

## Abstract

Myocardial fibrosis is a common feature of several heart diseases. The progressive deposition of extracellular matrix due to a persistent injury to cardiomyocytes may trigger a vicious cycle that leads to persistent structural and functional alterations of the myocardium. Some drugs (like renin–angiotensin–aldosterone system inhibitors) have been shown to reduce extracellular matrix deposition, but no primarily anti-fibrotic medications are currently used to treat patients with heart failure (HF). Pirfenidone is an oral antifibrotic agent approved for the treatment of idiopathic pulmonary fibrosis. Although its exact mechanism of action is not fully understood, pirfenidone might reduce the expression of profibrotic factors such as transforming growth factor-β (TGF-β), and proinflammatory cytokines, like tumor necrosis factor-α (TNF-α), interleukin (IL)-4, and IL-13, which could modulate the inflammatory response and inhibit collagen synthesis in lung tissue. There is some evidence that pirfenidone has antifibrotic activity in various animal models of cardiac disease. Furthermore, the positive results of the PIROUETTE trial, evaluating pirfenidone in patients with HF with preserved ejection fraction, have been very recently announced. This review summarizes the data about pirfenidone as a potential cardioprotective treatment.

Myocardial fibrosis is a common feature of several heart diseases, including heart failure (HF). Myocardial fibrosis is a compensatory mechanism that occurs to replace cardiomyocyte necrosis and preserve the structural integrity of the myocardium [1]. Nevertheless, progressive deposition of extracellular matrix (ECM) due to a persistent injury may trigger a vicious cycle leading to persistent structural and functional alterations of the myocardium. Thus, myocardial fibrosis has been considered a pharmacological target to prevent the development of clinical HF and slow down its progression. Although some drugs (like inhibitors of the renin/angiotensin/aldosterone system) have been shown to reduce ECM deposition, no primarily antifibrotic medications are used to treat patients with HF. These therapies are potentially interesting because they target a crucial disease mechanism and have little or no impact on hemodynamics.

Pirfenidone (5-methyl-1-phenyl-2-[1H]-pyridone) is an oral antifibrotic agent approved for the treatment of idiopathic pulmonary fibrosis (IPF) [2, 3]. Although its exact mechanism of action is not fully understood, pirfenidone might reduce the expression of profibrotic factors, such as transforming growth factor-β (TGF-β), and proinflammatory cytokines, like tumor necrosis factor-α (TNF-α), interleukin (IL)-4, and IL-13, which could modulate the inflammatory response and inhibit collagen synthesis in lung tissue [4, 5]. Several molecular pathways are involved in fibrotic tissue deposition by cardiac fibroblasts. Major profibrotic cell membrane receptors include those for connective tissue growth factor (CTGF), angiotensin-II, platelet-derived growth factor (PDGF), and TGF-β [6]. Indeed, multiple experimental studies have shown that pirfenidone had antifibrotic activity in various disease models [7], including pressure overload, diabetic and anthracycline-induced cardiomyopathies, myocardial infarction (MI), atrial fibrillation (AF), and Duchenne muscular dystrophy (Fig. 1).

**Fig. 1:**
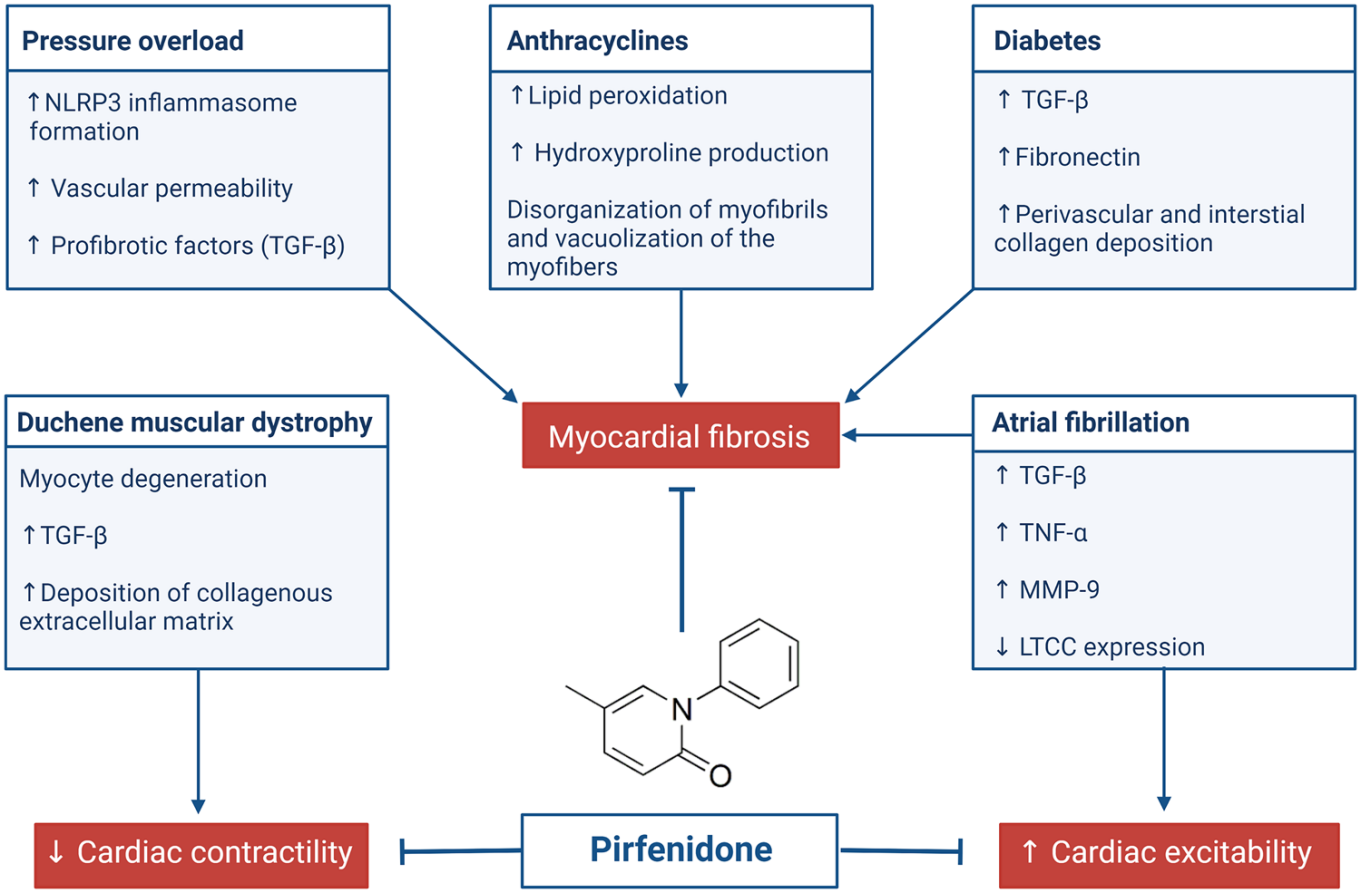
Cardiac protective effects of pirfenidone. See text for details. LTCC L-type calcium channel, MMP-9 matrix metalloproteinase 9, TGF-β transforming growth factor-beta, TNF-α tumor necrosis factor-alpha

This review summarizes the current evidence on the potential effects of pirfenidone in models of myocardial diseases and the recent findings about cardiac protection by pirfenidone in a clinical setting (Table 1). We searched the PubMed/Medline and EMBASE databases on June 15 and then again on September 10, 2021, using the terms: “pirfenidone AND (heart OR cardiac).” We also searched the https://clinicaltrials.gov/ website (last search on September 10, 2021). Given the design of this work as a narrative review, no formal criteria for study selection or appraisal were enforced.

**Table 1:** Results from preclinical and clinical studies

**Preclinical studies**			
**Study, ref**	**Model**	**Intervention**	**Findings**
Wang et al. [12]	TAC-induced mouse model of hypertension and LV hypertrophy	PFD (200 mg/kg) every 2 days from day 10 after surgery	- PDF increased survival rate and reduced fibroblast proliferation and the expression of TGF-β1 and hydroxyproline - PFD attenuated myocardial inflammation by regulating the NLRP3- inflammasome-mediated IL-1β signaling pathway
Mirkovic et al. [15]	Rat model of hypertensive cardiomyopathy obtained by uninephrectomy	DOCA-salt or no further treatment for 2 weeks PFD (0.4% in powdered rat food) for further 2 weeks	- PFD attenuated LV hypertrophy and reduced collagen deposition and diastolic stiffness
Yamazaki et al. [16]	Angiotensin II-induced mouse model of cardiac hypertrophy	PFD (300 mg/kg/day) for 2 weeks	- PFD inhibited angiotensin II-induce LV hypertrophy, decreased heart weight, attenuated mRNA expression of ANP, BNP, TGF β 1, and mineralocorticoid receptors
Yamagami et al. [17]	TAC-induced mouse model of hypertension and LV hypertrophy	PFD (400 mg/kg) twice daily from week 4 to week 8 after surgery	- PFD improved systolic function and suppressed LV dilation and fibrotic progression induced by pressure overload - PFD inhibited changes in the collagen 1 and Cldn5 expression levels resulting in reduced fibrosis and vascular permeability
Poble et al. [18]	Sugen/hypoxia rat model of severe pulmonary hypertension	PFD (30 mg/kg per day) 3 times a day for 3 weeks	- PFD reduced proliferation of pulmonary artery smooth cells and extracellular matrix deposition in lungs and RV
Andersen et al. [19]	Pressure overload RV failure rat model induced by pulmonary trunk banding	PFD (700 mg/kg/day) for 6 weeks	- PFD did not reduce RV fibrosis or improve RV hemodynamics
Miric et al. [22]	STZ rat model of diabetic cardiomyopathy	PFD (200 mg/day) from week 4 to week 8 after STZ treatment	- PFD attenuated LV perivascular and interstitial collagen deposition and diastolic stiffness increase induced by STZ - PFD did not normalize cardiac contractility
Giri et al. [24]	DXR-induced rat model of cardiac and renal toxicity	Saline + regular diet; DXR + regular diet; saline + the same diet mixed with 0.6% PFD; DXR + the same diet mixed with 0.6% PFD for 25 days	- PFD suppressed DXR-induced increases in hydroxyproline content in the heart and kidney, lipid peroxidation of the kidney and plasma, and protein content of the urine - PFD minimized the DXR-induced histopathological changes of heart and kidney
Li et al. [25]	Mice model of post-MI remodelling	PFD (300 mg/kg) by gavage daily for 4 weeks	- PFD inhibited the AT1R/p38 MAPK pathway, corrected the RAS imbalance and strongly enhanced heart LXR-α expression
Nguyen et al. [27]	Rat model of post-MI remodelling	PFD (1.2% in rat food) for 4 weeks from 1 week after surgery	- PFD decreased total and nonscar fibrosis, which correlated with decreased infarct scar, improved LV function and decreased ventricular tachycardia susceptibility
Adamo et al. [28]	- DT-induced mice model of myocardial injury - I/R-induced mice model of myocardial injury	PFD (0.5% in powdered rat food) from 3 days prior to DT-induced injury or I/R injury	- PFD attenuated LV remodelling and significantly improved survival rates - PFD had no effect on DT-induced cardiac myocyte cell death and on the number of neutrophils, monocytes or macrophages, but decreased CD19 + lymphocytes - The cardioprotective effects of PFD may rely upon a mechanism involving the modulation of cardiac B lymphocytes
Lee et al. [31]	Canine model of congestive heart failure	PFD (800 mg 3 times per day) for 3 weeks	- PFD attenuated arrhythmogenic left atrial remodeling, left atrial fibrosis, atrial fibrillation duration - PFD reduced TGF-β, TNF-a and metalloproteinase-9 and increased TIMP-4 levels
Van Erp et al. [36]	Dystrophin-deficient mdx mouse model of Duchenne muscular dystrophy	PFD for 7 months	- PFD improved cardiac contractility and decreased TGF-β expression but did not reduce extracellular matrix deposition
**Clinical studies**			
**Study, ref**	**Generalities**	**Intervention**	**Findings**
AlAnsari et al. [37]	- Single-center retrospective study - 124 patients with IPF (64 treated with PFD, 60 controls)		- PFD treatment did not improve parameters of LV structure, diastolic function, systolic function and global longitudinal strain
AlAnsari et al. [38]	- Single-center retrospective study - 24 patients with a history of HFpEF and IPF		- PFD was associated with decreases in indexed LV end diastolic and end systolic volumes - There were no significant changes in LV diastolic, systolic function and strain
Lewis et al. [39]	- Randomized, double-blind, placebo-controlled, phase 2 trial (PIROUETTE trial) - 94 patients with HFpEF (LV ejection fraction ≥ 45%) and myocardial fibrosis (ECM volume ≥ 27% measured by CMR) randomized to PFD (*n* = 47) or placebo (*n* = 47)	PFD for 52 weeks	- PFD was associated with a reduction in ECM volume and in log NT-proBNP compared to placebo - No significant differences in measures of diastolic function, 6-min walking distance nor KCCQ summary score values were observed

*ANP* atrial natriuretic peptide, *AT1R* angiotensin II type 1 receptor, *BNP* B-type natriuretic peptide, *CMR* cardiac magnetic resonance, *DOCA* deoxycorticosterone acetate, *DT* diphtheria toxin, *DXR* doxorubicin, *ECM* extracellular matrix, *HFpEF* heart failure with preserved ejection fraction, *I/R* ischemia–reperfusion, *IPF* idiopathic pulmonary fibrosis, *KCCQ* Kansas City Cardiomyopathy Questionnaire, *LV* left ventricle, *LXR-α* liver X receptor-α, *MI* myocardial infarction, *p38 MAPK* phospho-p38 mitogen-activated protein kinase, *PFD* pirfenidone, *RAS* renin-angiotensin system, *RV* right ventricle, *STZ* stretpozocin, *TAC* transverse aortic constriction

## Pharmacokynetics and mechanism of action of pirfenidone

Pirfenidone (5-methyl-1-phenyl-2-[1H]-pyridone) is a small synthetic molecule which is rapidly absorbed in the gastrointestinal tract and whose half-life is about 3 h [6, 8]. It is metabolized in the liver (mainly by CYP1A2) and mostly excreted as the metabolite 5-carboxy-pirfenidone, either through the urine (80%) or in the feces (20%). Although pirfenidone relieves the progression of pulmonary fibrosis and improves survival of these patients [2, 3], its mechanism of action is only incompletely understood. Several research groups have reported that pirfenidone inhibits fibroblast proliferation and collagen synthesis by interfering with TGF-β signaling [9] and other fibrogenic growth factors, such as platelet-derived growth factor (PDGF) and basic fibroblast growth factor (bFGF) [10]. Pirfenidone also upregulates several matrix metalloproteinases (MMPs) attenuating ECM accumulation [11]. Furthermore, pirfenidone modulates acute inflammation by reducing the expression of inflammatory cytokines, most notably TNF-α, IL-4, and IL-13, and by inhibiting the formation of the Nod-like receptor pyrin domain containing 3 (NLRP3) inflammasome, a protein complex responsible for the recognition of stress signals and involved in the onset and maintenance of inflammatory responses [6, 12]. Finally, pirfenidone might modulate the activity and proliferation of both T and B lymphocytes [6]. Since pulmonary and myocardial fibrosis share common molecular pathways [9], there is a growing interest in investing the role of pirfenidone in myocardial disease.

## Cardiac protection in animal models

### Pressure overload

Myocyte hypertrophy and myocardial fibrosis are two key features of hypertensive cardiomyopathy. The increased synthesis of collagen types I and III by cardiac fibroblasts in hypertensive hearts results in the deposition of fibrotic tissue which at earlier stages transmits the force generated by hypertrophied myocytes to the entire ventricle [13]. However, the excessive accumulation of fibrotic tissue is responsible for increased myocardial stiffness and diastolic dysfunction [1]. TGF-β plays a central role in this response since an increased TGF- β expression is associated with an increased synthesis of collagen type I and III and the administration of anti-TGF-β-neutralizing monoclonal antibody reduces fibroblast proliferation and fibrotic tissue deposition in pressure-overloaded rats [14]. Additionally, inflammatory cells infiltrate the perivascular spaces of hypertensive hearts, which suggests that pressure overload might induce the expression of inflammatory chemokines (such as monocyte chemoattractant protein-1 [MCP-1]) and trigger a stepwise process of acute inflammation followed by reactive fibrosis [13].

Multiple studies have shown that pirfenidone might reduce vascular permeability, in the acute phase, and the subsequent development of chronic fibrosis [12, 15–17]. Wang et al. investigated pirfenidone in a mouse model of hypertensive left ventricular remodeling, induced by transverse aortic constriction (TAC) [12]. They demonstrated that pirfenidone attenuated myocardial fibrosis by suppressing myocardial inflammation. In particular, pirfenidone reduced the expression of IL-1β and TGFβ1 by modulating the expression of NLRP3, a protein induced by pressure overload and involved in NLRP3-inflammasome formation. Administration of pirfenidone was also associated with reduced TAC-induced hypertrophy, assessed by echocardiography, and reduced TAC-induced thickening of the left ventricular (LV) wall, without reduction of blood pressure. Furthermore, mice treated with pirfenidone showed a higher survival rate compared to the control group [12]. Another study tested pirfenidone in a rat model of hypertensive cardiomyopathy, induced by unilateral nephrectomy followed by administration of salt and deoxycorticosterone acetate. A 2-week treatment with pirfenidone attenuated LV hypertrophy and reduced diastolic stiffness without lowering systolic blood pressure or reversing the increased vascular responses to norepinephrine [15]. Similarly, Yamazaki et al. studied a cardiac hypertrophic mouse model and found that early pirfenidone administration reduced LV hypertrophy and inhibited perivascular and interstitial tissue fibrosis induced by angiotensin II infusion [16]. These effects were accompanied by reduced expression of natriuretic peptides (i.e., atrial and brain natriuretic peptides), which are closely related to cardiac hypertrophy, and in the levels of TGF-β1 and MCP-1. Furthermore, pirfenidone inhibited the expression of genes encoding for mineralocorticoid receptors in the mouse heart, which suggested that it could prevent cardiac remodeling, partially via the inhibition of aldosterone signaling pathways [16]. Another study by Yamagami et al. investigated the effects of pirfenidone on cardiac fibrosis in a pressure-overloaded HF model, achieved by transverse aortic constriction [17]. They found that pirfenidone reduced TGFβ-induced collagen expression and increased claudin 5 expression, a tight junction protein that regulates vascular permeability. These effects resulted in reduced fibrosis and reduced serum albumin leakage into the interstitial space.

The role of pirfenidone in models of right ventricular (RV) pressure overload is controversial. Pirfenidone reduced RV fibrosis in a Sugen-hypoxia model of pulmonary hypertension [18]. This effect could have been indirect, because it might depend on reduced pulmonary vascular resistance by pirfenidone. Indeed, Andersen et al. found that pirfenidone did not reduce fibrosis or improve RV hemodynamics, when the RV pressure overload was induced by pulmonary artery banding in a rat model [19].

### Diabetic and anthracycline-induced cardiomyopathies

Diabetic cardiomyopathy is characterized by structural and functional abnormalities, including systolic and diastolic dysfunction and LV hypertrophy [20]. In addition to renin–angiotensin–aldosterone system activation, increased oxidative stress and advanced glycation end-products, hyperglycemia and hyperinsulinemia contribute to cardiac fibrosis by stimulating TGF-β1 expression, although the specific mechanisms remain elusive [21]. Therefore, pirfenidone might attenuate the deposition of fibrotic tissue in diabetic cardiomyopathy by interfering with TGF-β signaling. In a rat model of diabetic cardiomyopathy [22], streptozotocin administration promoted interstitial collagen deposition in the kidney and the aorta. In addition, streptozotocin increased LV fibrosis and diastolic stiffness and reduced the maximum positive inotropic responses to norepinephrine and a calcium sensitizer in papillary muscles [22]. Pirfenidone treatment reversed cardiac and renal fibrosis and improved diastolic function, but did not normalize cardiac contractility or renal function [22].

Cardiotoxicity is a well-recognized adverse effect of several cancer therapies, most notably anthracyclines, which are associated with early myocardial edema and subsequent fibrosis [23]. Giri et al. investigated the protective role of pirfenidone in a rat model of anthracycline-induced toxicity [24]. They infused rats with doxorubicin, then treated them with either pirfenidone or a regular diet. Pirfenidone attenuated the doxorubicin-induced increase in hydroxyproline content and the histopathological changes (disorganization of cardiac myofibrils and vacuolization of the myofibers) in the heart [24].

### Myocardial infarction

Cardiac fibrosis following MI represents a critical mechanism in the development of HF and may also act as a substrate for ventricular tachyarrhythmias [6, 25, 26]. Pirfenidone therapy was consistently reported to reduce fibrosis in a rat model of post-MI remodelling. Treatment was started 1 week after ischemia–reperfusion injury and continued for 4 weeks. Pirfenidone-treated rats showed smaller infarct scars compared to controls (8.9% of LV myocardium vs. 15.7%, *p* = 0.014), less total LV fibrosis (15% vs. 30%, *p* < 0.003), a reduced decline in LV ejection fraction (LVEF) over 4 weeks (8.6% vs. 24.3% in controls, *p* < 0.01), and lower rates of ventricular tachycardia inducibility (28.6% vs. 73.3%, *p* < 0.05) [27]. In another rat model of MI, pirfenidone administration by gavage for 4 weeks after permanent ligation left anterior descending artery reduced cardiac fibrosis and infarct size [25]. The cardioprotective effects could be largely explained by enhanced liver X receptor-α (LXR-α) expression regulating the feedback loop of the angiotensin II type 1 receptor (AT1R)/ phospho-p38 mitogen-activated protein kinase (p38 MAPK) and renin-angiotensin system (RAS) axis [25]. Similarly, in 2 different in vivo mice models of acute myocardial injury (damage by diphtheria toxin [DT] and closed-chest ischemia–reperfusion injury), pirfenidone-treated mice showed attenuated LV remodeling and significantly improved survival rates compared to controls (*p* = 0.03) [28]. Treatment with pirfenidone had no effect on DT-induced cardiac myocyte cell death and on the number of neutrophils, monocytes, or macrophages, but decreased CD19 + lymphocytes. B cell depletion abrogated the beneficial effects of pirfenidone. In vitro studies demonstrated that stimulation with lipopolysaccharide and extracts from necrotic cells activated B lymphocytes, and pirfenidone blunted this activation. The authors concluded that pirfenidone may exert cardioprotective effects through a mechanism involving the modulation of cardiac B lymphocytes [28].

### Atrial fibrillation

Interstitial fibrosis may act as a substrate for the development of AF, and patients with AF have more atrial fibrosis than patients in sinus rhythm [29, 30]. Several studies on animal models have shown that fibrosis prolonged conduction times, leading to the creation of macro-reentrant circuits that increase susceptibility to AF and maintains AF [29].

In a canine model of HF, Lee et al. found that pirfenidone treatment regulated the homeostasis of the atrial ECM by reducing TGFβ, TNF-α, and MMP-9 levels and increasing the levels of an endogenous cardio-specific inhibitor of MMP, TIMP-4 [31]. These changes resulted in a reduction in atrial fibrosis and AF vulnerability. Pirfenidone could also prevent AF regardless of TGFβ signaling by promoting atrial electrical remodeling. Indeed, in adult rat cardiomyocytes, chronic treatment with pirfenidone increased the expression of L-type calcium channels. These channels are typically downregulated in AF; therefore, their increased expression by pirfenidone prolongs both the action potential duration and the refractory period thus lowering susceptibility to AF [32].

### Duchenne muscular dystrophy

Patients with Duchenne muscular dystrophy often develop systolic and diastolic dysfunction and myocardial fibrosis, often progressing to clinical HF [33, 34]. In fact, a dystrophin deficiency causes myocyte degeneration and an increased deposition of ECM resulting in a progressive impairment of cardiac function [35]. Van Erp et al. randomized 36 dystrophin-deficient mice to pirfenidone or placebo for 7 months. Pirfenidone ameliorated cardiac contractility and reduced TGF-β expression, but these effects were not associated with a reduction in myocardial fibrosis [36]. These findings corroborate the notion that pirfenidone might improve cardiac function by inhibiting the synthesis of inflammatory cytokines and reducing oxidative stress, regardless of its antifibrotic effect.

## Evidence of cardiac protection from clinical studies

The effects of pirfenidone on echocardiographic parameters of LV function were evaluated by two clinical studies. In the first one, pirfenidone treatment did not improve parameters of LV structure, diastolic function, systolic function and global longitudinal strain [37]. In the second one, pirfenidone was associated with decreases in indexed LV end diastolic and end systolic volumes, although no significant changes in LV diastolic, systolic function, and strain were observed [38]. However, both studies included only IPF patients and were limited by their small size and retrospective design.

To date, only one randomized, double-blind, placebo-controlled trial included patients with a cardiac condition. The Efficacy and Safety of Pirfenidone in Patients With Heart Failure and Preserved Left Ventricular Ejection Fraction (PIROUETTE) phase 2 trial evaluated the safety and efficacy of a 52-week treatment with pirfenidone in 94 patients with HF with preserved ejection fraction (LV ejection fraction ≥ 45%) and myocardial fibrosis (defined as an ECM volume ≥ 27% measured by cardiac magnetic resonance [CMR]) [39]. At 52 weeks, the extracellular volume displayed an absolute decrease by 0.7% in the pirfenidone group and an increase by 0.5% in the placebo group, with a between-group difference that was very small (also considering the variability in extracellular volume measurements by CMR), but still achieved statistical significance (–1.21%; 95% confidence interval, –2.12 to –0.31; *p* = 0.009). A limited but significant reduction in N-terminal pro-B-type natriuretic peptide values was also found. Conversely, no significant differences in measures of diastolic function, 6-min walking distance nor Kansas City Cardiomyopathy Questionnaire summary score values were observed [39]. These findings suggested that pirfenidone may be beneficial but further trials are needed to determine the clinical effectiveness and safety in a broader population.

## Conclusions

Pirfenidone is an antifibrotic drug that mostly studied in lung models, and its beneficial effects have been confirmed in clinical trials for the treatment of idiopathic pulmonary fibrosis. Given the important role of fibrosis in the pathophysiology of several cardiac disorders, an intriguing perspective is to repurpose pirfenidone as a treatment for cardiac disorders. Although there are some data showing that pirfenidone has antifibrotic effects in animal models of cardiac disease, evidence in human is currently limited to a phase 2 study evaluating a surrogate endpoint, namely, changes in extracellular volume on repeated CMR scans. Therefore, there is a crucial need for further studies on the safety and efficacy of pirfenidone for the treatment of cardiac disease in humans.
